# Supplementary material for: Sexual unfaithfulness can be judged with some accuracy from men's but not women's faces
Source: R Soc Open Sci. 2019 Apr 17;6(4):181552. doi: 10.1098/rsos.181552 (PMC6502397; doi:10.1098/rsos.181552)
Supplement: Supplementary_material.docv [file rsos181552supp1.docx]

**Supplementary material**

**Sexual unfaithfulness can be judged with some accuracy from men’s but not women’s faces**

Yong Zhi Foo^1,2,*^, Antonina Loncarevic^1^, Leigh W. Simmons^1,2^, Clare A. M. Sutherland^1^, Gillian Rhodes^1^

^1^ARC Centre of Excellence in Cognition and its Disorders, School of Psychological Sciences, University of Western Australia, 35 Stirling Hwy, Crawley, 6009, WA, Australia

^2^Centre for Evolutionary Biology & School of Biological Sciences, University of Western Australia, 35 Stirling Hwy, Crawley, 6009, WA, Australia

*Author for correspondence (E-mail: yong.foo@uwa.edu.au, Tel.: +61 (8) 6488 3240).

Table S1. Zero-order correlations, *p*-values, and *N*s for age, cheating and poaching scores and facial impression ratings of men’s faces. Because the cheating and poaching data could not be transformed to normality, we present parametric Pearson’s R above the diagonal and non-parametric Kendall’s Tau below. Outliers in these two variables were winsorized to 3SD above the mean (2 men’s cheat values; 3 men’s poach values) (Field, 2009)

|  | Age | Cheat | Poach | Unfaithfulness rated by men | Unfaithfulness rated by women | Untrustworthiness | Attractiveness | Sexual dimorphism |
| --- | --- | --- | --- | --- | --- | --- | --- | --- |
| Age |  | .30 | .34 | .13 | .20 | .10 | -.32 | .43 |
|  |  | .002 | .000 | .203 | .049 | .324 | .002 | .000 |
|  |  | 101 | 101 | 101 | 101 | 101 | 98 | 98 |
| Cheat | .28 |  | .69 | .19 | .25 | -.05 | -.02 | .24 |
|  | .000 |  | .000 | .059 | .013 | .638 | .854 | .017 |
|  | 101 |  | 101 | 101 | 101 | 101 | 98 | 98 |
| Poach | .26 | .38 |  | .18 | .24 | .09 | -.14 | .16 |
|  | .001 | .000 |  | .065 | .014 | .385 | .179 | .115 |
|  | 101 | 101 |  | 101 | 101 | 101 | 98 | 98 |
| Unfaithfulness rated by men | .18 | .11 | .13 |  | .96 | .31 | .38 | .62 |
|  | .009 | .163 | .100 |  | .000 | .001 | .000 | .000 |
|  | 101 | 101 | 101 |  | 101 | 101 | 98 | 98 |
| Unfaithfulness rated by women | .24 | .13 | .19 | .82 |  | .29 | .38 | .67 |
|  | .001 | .101 | .014 | .000 |  | .003 | .000 | .000 |
|  | 101 | 101 | 101 | 101 |  | 101 | 98 | 98 |
| Untrustworthiness | .02 | -.07 | .04 | .20 | .18 |  | -.41 | .24 |
|  | .726 | .374 | .602 | .003 | .009 |  | .000 | .017 |
|  | 101 | 101 | 101 | 101 | 101 |  | 98 | 98 |
| Attractiveness | -.10 | .00 | -.07 | .23 | .23 | -.29 |  | .22 |
|  | .165 | .988 | .417 | .001 | .001 | .000 |  | .032 |
|  | 98 | 98 | 98 | 98 | 98 | 98 |  | 98 |
| Sexual dimorphism | .35 | .13 | .12 | .42 | .46 | .14 | .14 |  |
|  | .000 | .093 | .130 | .000 | .000 | .045 | .039 |  |
|  | 98 | 98 | 98 | 98 | 98 | 98 | 98 |  |

Table S2. Zero-order correlations (Pearson’s R above the diagonal and Kendall’s Tau below), *p*-values, and *N* for age, cheating and poaching scores and facial impression ratings of women’s faces. Outliers in these two variables were winsorized to 3SD above the mean (1 woman’s cheat values; 1 woman’s poach values) (Field, 2009)

|  | Age | Cheat | Poach | Unfaithfulness rated by men | Unfaithfulness rated by women | Trustworthiness | Attractiveness | Sexual dimorphism |
| --- | --- | --- | --- | --- | --- | --- | --- | --- |
| Age |  | .12 | .15 | -.30 | -.22 | .13 | -.26 | -.11 |
|  |  | .268 | .163 | .005 | .037 | .229 | .013 | .301 |
|  |  | 88 | 88 | 88 | 88 | 88 | 88 | 88 |
| Cheat | .17 |  | .19 | .06 | .05 | .16 | .00 | -.10 |
|  | .055 |  | .078 | .561 | .675 | .144 | .971 | .340 |
|  | 88 |  | 88 | 88 | 88 | 88 | 88 | 88 |
| Poach | .04 | .14 |  | .07 | .07 | -.07 | .04 | .01 |
|  | .677 | .162 |  | .528 | .524 | .522 | .724 | .928 |
|  | 88 | 88 |  | 88 | 88 | 88 | 88 | 88 |
| Unfaithfulness rated by men | -.19 | .01 | .11 |  | .94 | .12 | .77 | .64 |
|  | .012 | .943 | .213 |  | .000 | .264 | .000 | .000 |
|  | 88 | 88 | 88 |  | 88 | 88 | 88 | 88 |
| Unfaithfulness rated by women | -.13 | .00 | .12 | .79 |  | .28 | .67 | .54 |
|  | .093 | .993 | .162 | .000 |  | .008 | .000 | .000 |
|  | 88 | 88 | 88 | 88 |  | 88 | 88 | 88 |
| Trustworthiness | .08 | .17 | -.05 | .11 | .22 |  | -.28 | -.25 |
|  | .280 | .044 | .547 | .123 | .002 |  | .007 | .017 |
|  | 88 | 88 | 88 | 88 | 88 |  | 88 | 88 |
| Attractiveness | -.20 | -.04 | .08 | .60 | .50 | -.14 |  | .80 |
|  | .008 | .662 | .354 | .000 | .000 | .061 |  | .000 |
|  | 88 | 88 | 88 | 88 | 88 | 88 |  | 88 |
| Sexual dimorphism | -.04 | -.08 | .02 | .46 | .36 | -.17 | .59 |  |
|  | .582 | .329 | .817 | .000 | .000 | .018 | .000 |  |
|  | 88 | 88 | 88 | 88 | 88 | 88 | 88 |  |
